# Synthesis and structure of 4-(benz­yloxy)phenyl 3,5-di­methyl­benzoate

**DOI:** 10.1107/S2056989026007474

**Published:** 2026-07-28

**Authors:** P. Roopadevi, G. N. Venkata Reddy, B. Bommalingaiah, H. C. Devarajegowda, B. S. Palakshamurthy

**Affiliations:** a Department of Physics Yuvaraja's College University of Mysore,Mysore-570005 Karnaataka India; b Department of Physics, Government Science College, Chithradurga-577501, Kanataka, India; c Department of PG Studies and Research in Physics Albert Einstein Block UCS Tumkur University, Tumkur Karnataka-572103 India; University of Aberdeen, United Kingdom

**Keywords:** crystal structure, Hirshfeld surface, (benz­yloxy)phen­yl, 3,5-di­methyl­benzolate

## Abstract

In the title compound, C_22_H_20_O_3_, the dihedral angles between the central and peripheral aromatic rings are 83.47 (2) and 66.00 (2)° and the packing is consolidated by C—H⋯π inter­actions. Hirshfeld surface analysis indicates that the major contributions to the two-dimensional fingerprint plots arise from H⋯H (51.1%), C⋯H/H⋯C (32.7%), and H⋯O/O⋯H (14.3%) contacts. Energy framework calculations indicate that dispersion energy makes the largest contribution (–240 kJ mol^−1^) to the packing compared to the other energy components.

## Abstract 2

In the title compound, the dihedral angles between the central and peripheral aromatic rings are 83.47 (2) and 66.00 (2)° and the packing is consolidated by C—H⋯π inter­actions.

## 1. Chemical context

Benz­yloxy-substituted aromatic compounds attract considerable attention because of their broad spectrum of biological activities including anti­malarial, anti­bacterial, and anti­platelet properties (Mohebi *et al.*, 2022 ▸; de Candia *et al.*, 2009 ▸). Other benz­yloxy derivatives act as hypolipidemic, anti­oxidant and hypoglycemic agents and significantly influence lipid metab­olism and insulin resistance (Hassan *et al.*, 2021 ▸; Kuranov *et al.*, 2020 ▸). In addition, benz­yloxy-containing quinolone derivatives display anti­tumour activity against several human cancer cell lines (Chen *et al.*, 2015 ▸). Phenyl benzoate analogues also show notable anti­microbial activity against both Gram-positive and Gram-negative bacteria (Thawkar *et al.*, 2022 ▸), whereas methyl benzoate congeners exhibit repellent and fumigant activities against stored-product pests (Xiao *et al.*, 2024 ▸).
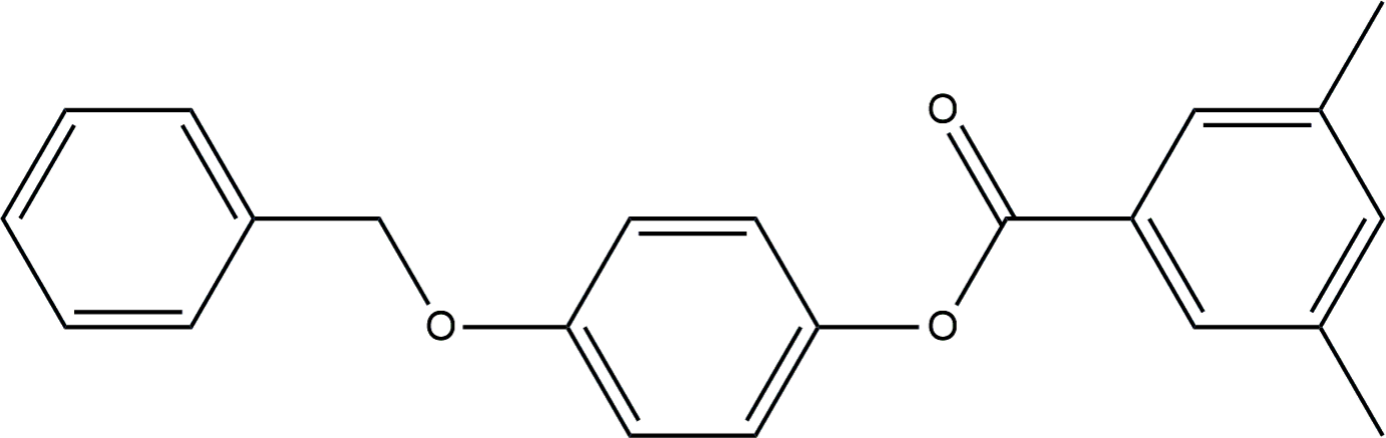


As part of our studies in this area, we now describe the synthesis and structure of the title compound, C_22_H_20_O_3_ (**I**).

## 2. Structural commentary

**Figure 1:**
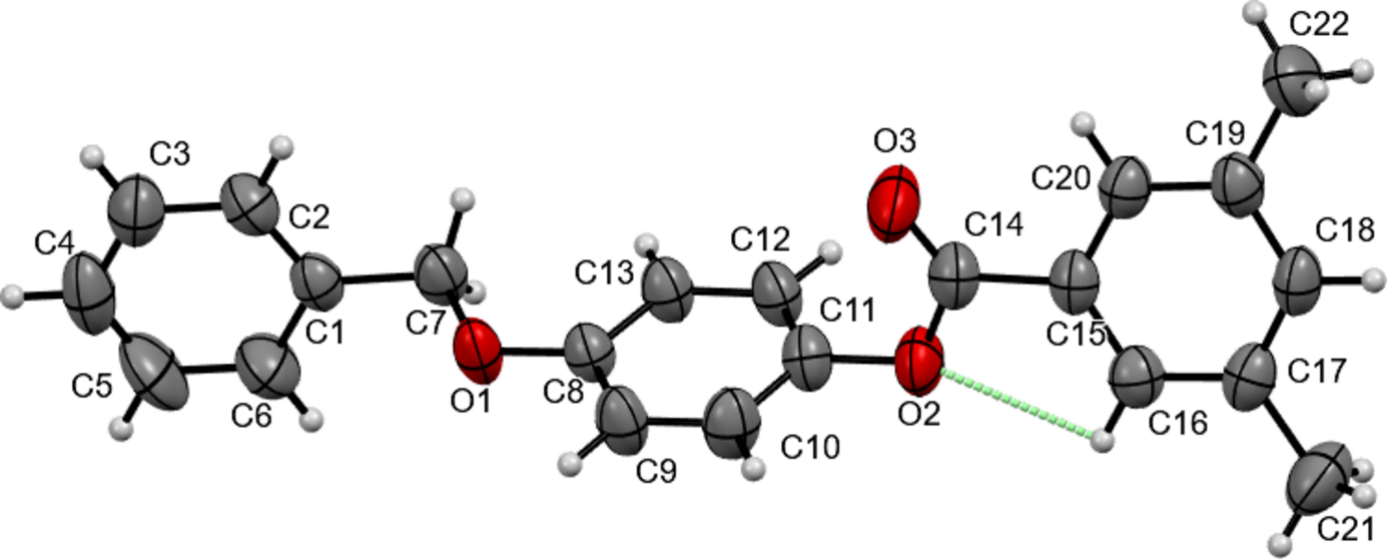
The mol­ecular structure of (**I**) with 50% probability ellipsoids and the intra­molecular hydrogen bond shown as a green dashed line.

Compound (**I**) crystallizes in the monoclinic crystal system in space group *P*2_1_/*c* with one mol­ecule in the asymmetric unit (Fig. 1 ▸). The dihedral angles between the central phenol ring (C8–C13) and pendant benz­yloxy phenyl (C1–C6) and di­methyl­benzoate rings (C15–C20) are 83.47 (2) and 66.00 (2)°, respectively, reflecting a markedly twisted mol­ecular geometry; the dihedral angle between the outer rings is 18.1 (2)°. The steric preference of the mol­ecule is further governed by the conformation of the ether linkage: the C1—C7—O1—C8 torsion angle is −177.1 (2)°, indicating an *anti* arrangement, presumably to minimise steric repulsion between the adjacent aromatic fragments. Two short intra­molecular C—H⋯O contacts (Table 1 ▸) are observed. The overall mol­ecular architecture of (**I**) is therefore characterized by the non-coplanar disposition of the aromatic rings linked through nearly *anti*-oriented ether and ester functionalities.

## 3. Supra­molecular features

**Figure 2:**
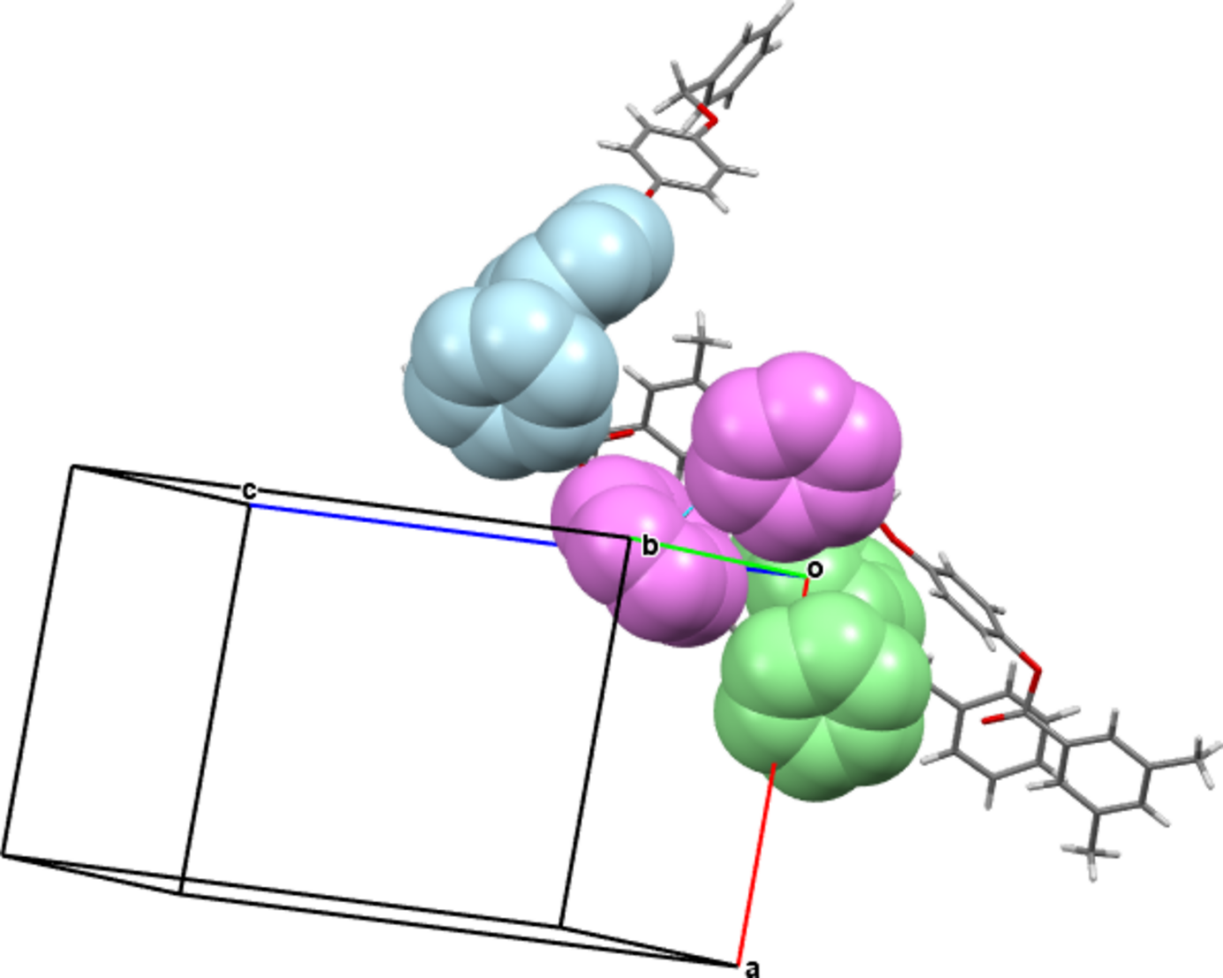
The mol­ecular packing showing edge-to-face EDDIE. The EDDIEs are at three positions in the mol­ecule – light blue (C18—H18⋯*Cg*3 interactions), pink (C3—H3⋯*Cg*2 interactions) and green (C13—H13⋯*Cg*1 interactions).

In the extended structure of (**I**), aromatic C—H⋯π inter­actions are observed. Modern theoretical and experimental studies have demonstrated that an electrostatic inter­pretation for this type of bond is an oversimplification. Rather than arising from a distinct attractive inter­action between π orbitals, the stability of aromatic assemblies is governed by a delicate balance of electrostatic inter­actions, dispersion forces, desolvation, induction, and exchange repulsion (EDDIE; Xiao *et al.*, 2026 ▸). The aromatic C—H moieties facing towards the centroid of the aromatic rings of neighbouring mol­ecules generate edge-to-face (T-shaped) stacking, namely C3—H3⋯*Cg*2 (between pink-coloured rings), C13—H13⋯*Cg*1 (between green-coloured rings) and C18—H18⋯*Cg*3 (between light-blue-coloured rings) as shown in Fig. 2 ▸, where *Cg*1, *Cg*2 and *Cg*3 are the centroids of the C1–C6, C8–C13 and C15–C20 rings, respectively (Table 1 ▸).

## 4. Hirshfeld surface analysis

**Figure 3:**
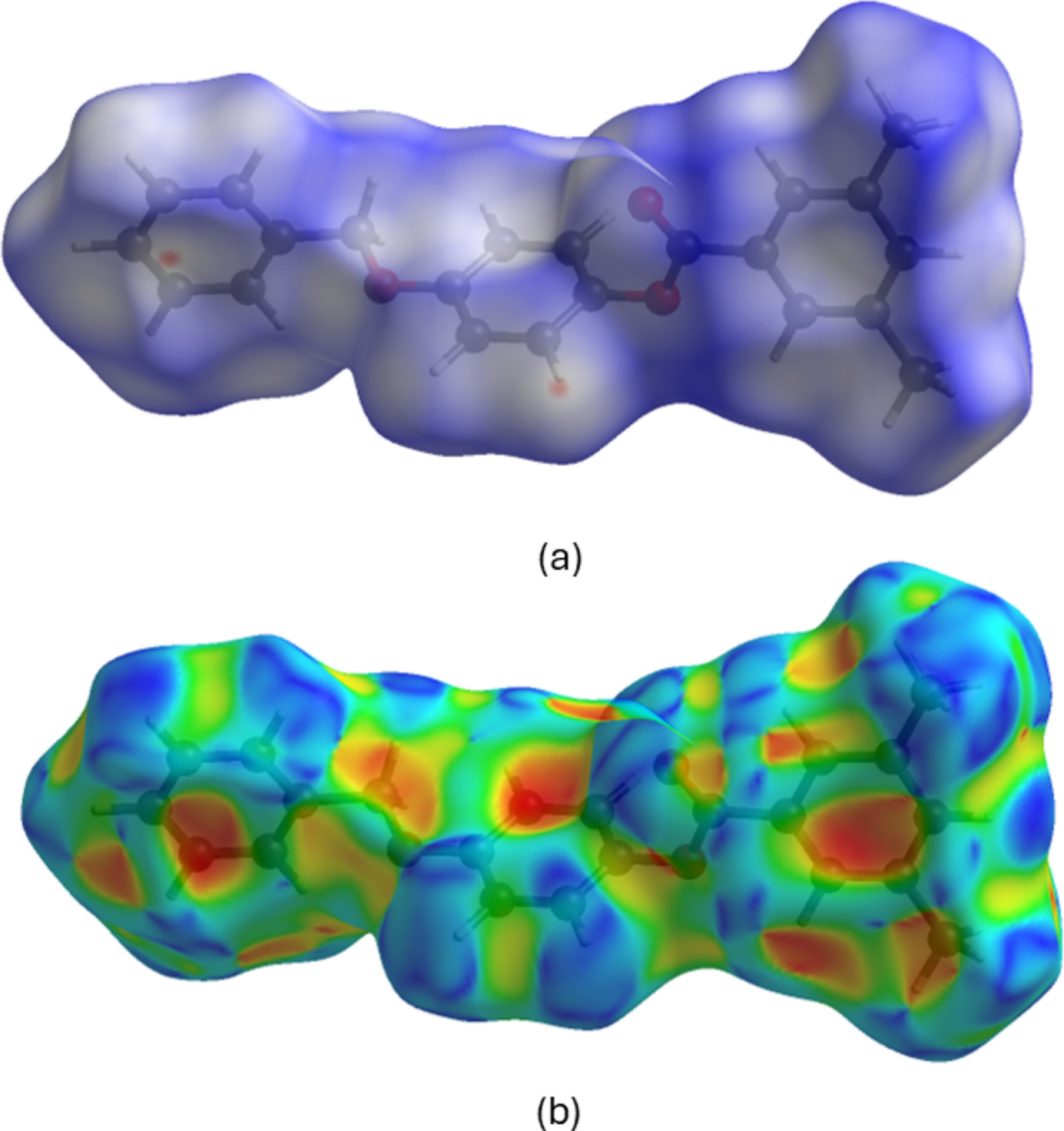
The Hirshfeld surface view plotted over (*a*) *d*_norm_ and (*b*) shape-index.

**Figure 4:**
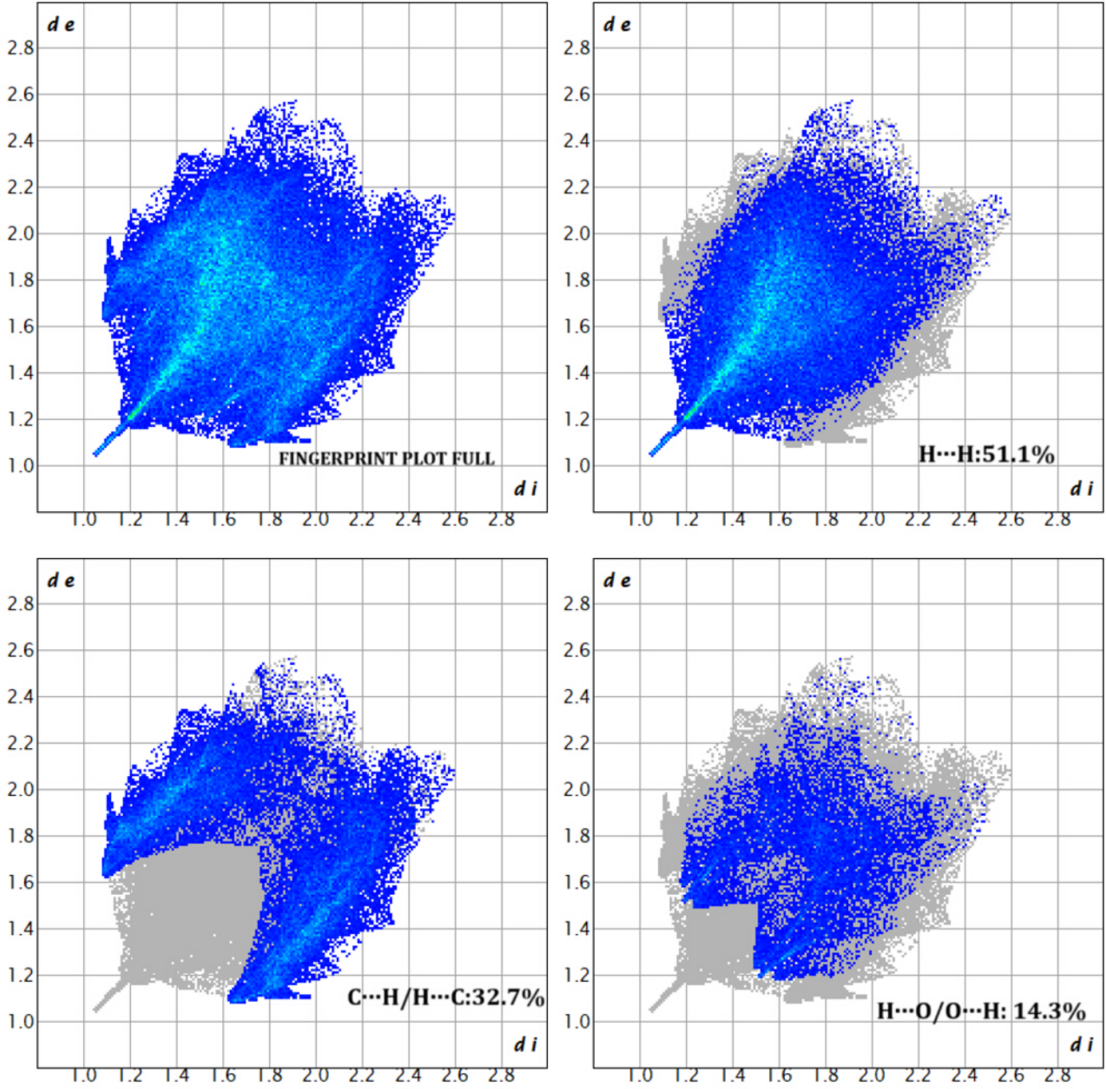
The two-dimensional fingerprint plots showing different contact types.

A Hirshfeld surface analysis was carried out using *Crystal Explorer 17.5* (Spackman *et al.*, 2021 ▸) to further qu­antify the inter­molecular inter­actions. The three-dimensional Hirshfeld surfaces plotted over *d*_norm_ and shape-index are shown in Fig. 3 ▸*a*. The red spots around the aromatic rings of the mol­ecule signifies the presence of centroids which are shown in Fig. 3 ▸*b*. The two-dimensional fingerprint plots indicate that the most prominent contributions for the Hirshfeld surfaces are from H⋯H (51.1%), C⋯H/H⋯C (32.7%) and H⋯O/O⋯H (14.3%), as shown in Fig. 4 ▸.

A crystal void analysis was performed using a 0.002 a.u. electron-density isosurface. The calculated void volume and surface area were found to be 223.4 Å^3^ and 770.7 Å^2^, respectively. The voids occupy 12.5% of the unit-cell volume, indicating a relatively efficient crystal packing with limited empty space within the volume. The large surface area implies enhanced potential for non-covalent inter­actions, which appear to play a key role in consolidating the packing.

**Figure 5:**
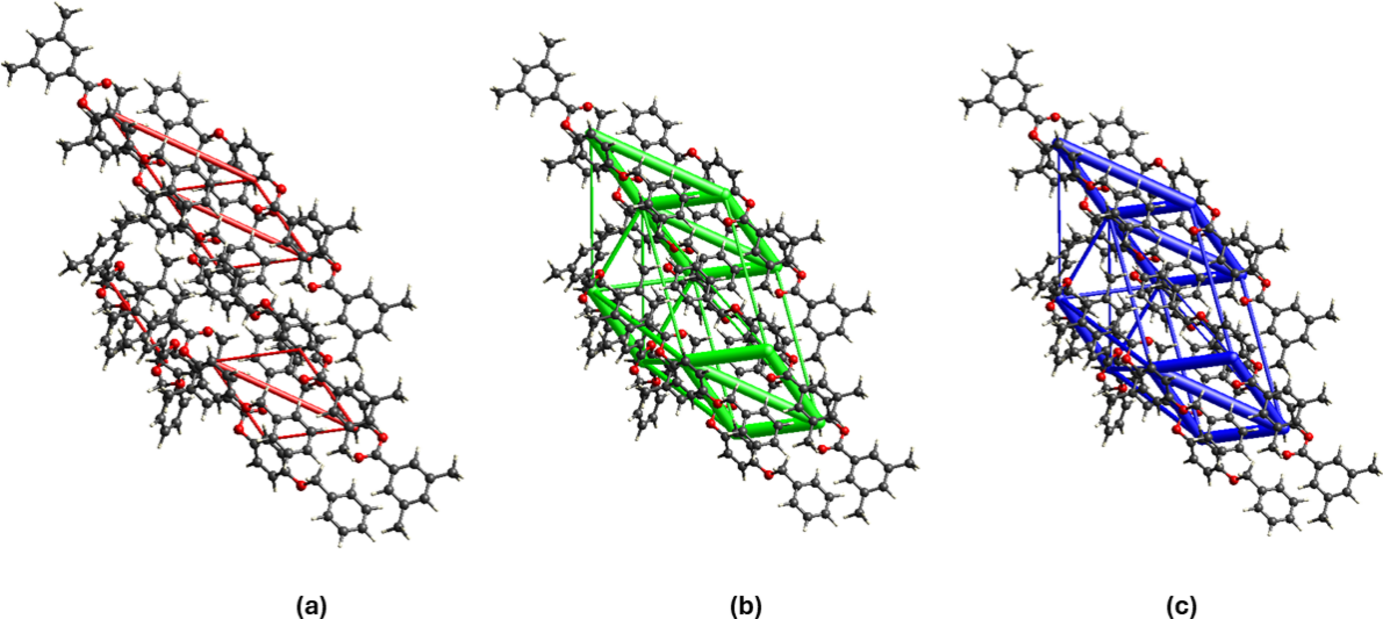
The energy framework topology generated for the (*a*) Coulombic, (*b*) dipersion and (*c*) total inter­action energies.

Inter­action energies for the mol­ecule were calculated using the basis set B3LYP\631-G(d,p) for mol­ecular pairs within a cluster of 3.8 Å radius, giving *E*_ele_= −49.7 kJ mol^−1^, *E*_pol_ = −15.4 kJ mol^−1^, *E*_dis_ = −240.1 kJ mol^−1^ and *E*_rep_ = 117.1 kJ mol^−1^. The energy framework is shown in Fig. 5 ▸.

## 5. Database survey

A search of the Cambridge Structural Database (CSD, version 6.01, March 2026; Groom *et al.*, 2016 ▸) for structures containing the 4-(benz­yloxy)phenyl fragment gave 176 hits. In all of these structures, the benz­yloxy substituent adopts an extended conformation, with torsion angles for the Ar—CH_2_—O—Ar linkage lying in the range 165–180°, which is in agreement with the torsion angle observed in (**I**). The dihedral angle between the benz­yloxy phenyl ring and the adjacent aromatic ring varies considerably, ranging from 1.23° in (*E*)-1-(4-(benz­yloxy)phen­yl)-3-(4-hy­droxy­phen­yl)prop-2-en-1-one (CSD refcode GIDBIG; Ramkumar *et al.*, 2013 ▸) and 9.61° in (*E*)-3-[4-(benz­yloxy)phen­yl]-1-(4-hy­droxy­phen­yl)prop-2-en-1-one (GIDBOM; Ramkumar *et al.*, 2013 ▸) to 87.69° in (2*E*)-3-[4-(benz­yloxy)phen­yl]-1-(pyridin-3-yl)prop-2-en-1-one (QEDCEJ; Fun *et al.*, 2012 ▸), indicating that the relative orientation of the aromatic rings is strongly influenced by the nature of the substituents and crystal-packing effects. A separate search for structures containing the 3,5-di­methyl­benzoate fragment yielded 17 entries, among which 3-*tert*-butyl-4-oxo-3,4-di­hydro­phthalazin-1-yl 3,5-di­methyl­ben­zo­ate (XISHEN; Wu *et al.*, 2008 ▸) was found to be the most closely related to (**I**). The aromatic ring systems in XISHEN are nearly orthogonal, with dihedral angles of 87.2 and 89.1°, similar to the equivalent values observed in (**I**). These observations indicate that the benz­yloxy ether linkage favours an extended *anti* conformation, whereas the orientations of the aromatic rings are determined by steric and packing requirements.

## 6. Synthesis and crystallization

A mixture of 3,5-di­methyl­benzoic acid (0.158 g, 1.052 mmol), 4-benz­yloxy-phenol (0.200 g, 0.999 mmol), *N*,*N*′-dicyclohexylcarbodiimide (DCC; 0.177 g, 0.8610 mmol) and a catalytic qu­antity of di­methyl­amino­pyrimidine was stirred in dry di­chloro­methane at room temperature for about 12 h. The reaction mixture was filtered to remove the insoluble byproduct di­cyclo­hexyl­urea. The solvent was removed and the residue purified by column chromatography on silica gel using di­chloro­methane as the mobile phase. Removal of solvent afforded a residue which was recrystallized from di­chloro­methane solution to afford crystals for single-crystal X-ray studies. Off-white solid; yield 82%. IR (KBr), ν_max_ (cm^−1^) : 2928, 2850, 1715, 1456, 1260, 1171, 821; ^1^H NMR (500 MHz, CDCl_3_, δ/ppm): 7.83 (*d*, 2H, *J* = 7.5 Ar-H), 7.62 (*m*, 1H, Ar-H), 7.52–7.04 (*m*, 9H, Ar-H), 5.12 (*s*, 2H, Ar-CH_2_O^−^), 2.34 (*t*, 6H, 2 × –CH_3_). Elemental analysis (%) calculated: C 79.50; H 6.07; O 14.44 and experimentally found: C 79.53; H 6.15.

## 7. Refinement

Crystal data, data collection and structure refinement details are summarized in Table 2 ▸. All the hydrogen atoms were located from difference maps and refined isotropically using a riding model with C—H = 0.93–0.97 Å and *U*_iso_(H) = 1.2*U*_eq_(C) or 1.5*U*_eq_ (methyl C).

## Supplementary Material

Crystal structure: contains datablock(s) I. DOI: 10.1107/S2056989026007474/hb8241sup1.cif

Structure factors: contains datablock(s) I. DOI: 10.1107/S2056989026007474/hb8241Isup2.hkl

Supporting information file. DOI: 10.1107/S2056989026007474/hb8241Isup3.cml

CCDC reference: 2574792

Additional supporting information:  crystallographic information; 3D view; checkCIF report

## Figures and Tables

**Table 1:** Hydrogen-bond geometry (Å, °) *Cg*1, *Cg*2 and *Cg*3 are the centroids of the C1–C6, C8–C13 and C15–C20 rings, respectively.

*D*—H⋯*A*	*D*—H	H⋯*A*	*D*⋯*A*	*D*—H⋯*A*
C16—H16⋯O2	0.93	2.47	2.764 (2)	99
C20—H20⋯O3	0.93	2.54	2.832 (2)	98
C3—H3⋯*Cg*2^i^	0.93	2.73	3.592 (3)	154
C13—H13⋯*Cg*1^ii^	0.93	2.98	3.794 (3)	147
C18—H18⋯*Cg*3^iii^	0.93	2.95	3.821 (3)	156

Symmetry codes: (i) 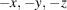; (ii) 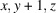; (iii) 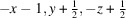.

**Table 2:** Experimental details

Crystal data	
Chemical formula	C_22_H_20_O_3_
*M*_r_	332.38
Crystal system, space group	Monoclinic, *P*2_1_/*c*
Temperature (K)	278
*a*, *b*, *c* (Å)	11.0755 (5), 6.7138 (3), 24.0578 (13)
β (°)	90.074 (2)
*V* (Å^3^)	1788.90 (15)
*Z*	4
Radiation type	Mo *K*α
μ (mm^−1^)	0.08
Crystal size (mm)	0.42 × 0.32 × 0.25

Data collection	
Diffractometer	Bruker SMART APEXII CCD
Absorption correction	Multi-scan (*SADABS*; Krause et al., 2015 ▸)
*T*_min_, *T*_max_	0.964, 0.984
No. of measured, independent and observed [*I* > 2σ(*I*)] reflections	23611, 3830, 2707
*R*_int_	0.061
(sin θ/λ)_max_ (Å^−1^)	0.636

Refinement	
*R*[*F*^2^ > 2σ(*F*^2^)], *wR*(*F*^2^), *S*	0.069, 0.158, 1.05
No. of reflections	3830
No. of parameters	229
H-atom treatment	H-atom parameters constrained
Δρ_max_, Δρ_min_ (e Å^−3^)	0.16, −0.17

Computer programs: *APEX2* and *SAINT* (Bruker, 2017 ▸), *SHELXT2018/3* (Sheldrick, 2015*a* ▸), *SHELXL2019/2* (Sheldrick, 2015*b* ▸), *Mercury* (Macrae *et al.*, 2020 ▸) and *publCIF* (Westrip,2010 ▸).

## supplementary crystallographic information

### 4-(Benzyloxy)phenyl 3,5-dimethylbenzoate Crystal data

**Table:**

C_22_H_20_O_3_	*F*(000) = 704
*M**_r_* = 332.38	*D*_x_ = 1.234 Mg m^−^^3^
Monoclinic, *P*2_1_/*c*	Mo *K*α radiation, λ = 0.71073 Å
Hall symbol: -P 2ybc	Cell parameters from 2707 reflections
*a* = 11.0755 (5) Å	θ = 3–26°
*b* = 6.7138 (3) Å	µ = 0.08 mm^−^^1^
*c* = 24.0578 (13) Å	*T* = 278 K
β = 90.074 (2)°	Prism, colourless
*V* = 1788.90 (15) Å^3^	0.42 × 0.32 × 0.25 mm
*Z* = 4	

### 4-(Benzyloxy)phenyl 3,5-dimethylbenzoate Data collection

**Table:**

Bruker SMART APEXII CCD diffractometer	3830 independent reflections
Radiation source: fine-focus sealed tube	2707 reflections with *I* > 2σ(*I*)
Graphite monochromator	*R*_int_ = 0.061
Detector resolution: 1.09 pixels mm^-1^	θ_max_ = 26.9°, θ_min_ = 3.5°
φ and Ω scans	*h* = −14→14
Absorption correction: multi-scan (SADABS; Krause et al., 2015)	*k* = −8→8
*T*_min_ = 0.964, *T*_max_ = 0.984	*l* = −30→30
23611 measured reflections	

### 4-(Benzyloxy)phenyl 3,5-dimethylbenzoate Refinement

**Table:**

Refinement on *F*^2^	Primary atom site location: structure-invariant direct methods
Least-squares matrix: full	Secondary atom site location: difference Fourier map
*R*[*F*^2^ > 2σ(*F*^2^)] = 0.069	Hydrogen site location: inferred from neighbouring sites
*wR*(*F*^2^) = 0.158	H-atom parameters constrained
*S* = 1.05	*w* = 1/[σ^2^(*F*_o_^2^) + (0.0517*P*)^2^ + 0.8532*P*] where *P* = (*F*_o_^2^ + 2*F*_c_^2^)/3
3830 reflections	(Δ/σ)_max_ < 0.001
229 parameters	Δρ_max_ = 0.16 e Å^−^^3^
0 restraints	Δρ_min_ = −0.17 e Å^−^^3^
0 constraints	

### 4-(Benzyloxy)phenyl 3,5-dimethylbenzoate Special details

**Table:**

Geometry. All esds (except the esd in the dihedral angle between two l.s. planes) are estimated using the full covariance matrix. The cell esds are taken into account individually in the estimation of esds in distances, angles and torsion angles; correlations between esds in cell parameters are only used when they are defined by crystal symmetry. An approximate (isotropic) treatment of cell esds is used for estimating esds involving l.s. planes.

### 4-(Benzyloxy)phenyl 3,5-dimethylbenzoate Fractional atomic coordinates and isotropic or equivalent isotropic displacement parameters (Å^2^)

**Table:**

	*x*	*y*	*z*	*U*_iso_*/*U*_eq_	
O1	0.12931 (15)	0.1623 (2)	0.08486 (7)	0.0570 (5)	
O2	−0.12798 (15)	0.7774 (2)	0.18195 (7)	0.0608 (5)	
C1	0.2390 (2)	−0.0041 (3)	0.01391 (9)	0.0479 (5)	
C16	−0.2601 (2)	1.0686 (3)	0.23572 (10)	0.0520 (6)	
H16	−0.197453	1.007649	0.255106	0.062*	
C15	−0.29629 (19)	0.9927 (3)	0.18481 (9)	0.0480 (5)	
C11	−0.0601 (2)	0.6240 (3)	0.15647 (10)	0.0525 (6)	
C8	0.0718 (2)	0.3243 (3)	0.10781 (10)	0.0484 (5)	
C7	0.1882 (2)	0.1918 (3)	0.03282 (10)	0.0565 (6)	
H7A	0.252537	0.288696	0.036851	0.068*	
H7B	0.130991	0.241637	0.005595	0.068*	
C20	−0.3896 (2)	1.0829 (3)	0.15600 (10)	0.0531 (6)	
H20	−0.414548	1.030152	0.122143	0.064*	
C12	0.0072 (2)	0.6637 (3)	0.11043 (10)	0.0561 (6)	
H12	0.008638	0.791875	0.095795	0.067*	
O3	−0.27559 (18)	0.7290 (3)	0.11930 (9)	0.0817 (6)	
C18	−0.4082 (2)	1.3223 (4)	0.22797 (10)	0.0570 (6)	
H18	−0.446272	1.434124	0.242588	0.068*	
C14	−0.2366 (2)	0.8194 (3)	0.15803 (11)	0.0545 (6)	
C13	0.0736 (2)	0.5138 (3)	0.08533 (10)	0.0538 (6)	
H13	0.118923	0.540372	0.053671	0.065*	
C19	−0.4461 (2)	1.2503 (4)	0.17688 (10)	0.0557 (6)	
C9	0.0071 (2)	0.2875 (3)	0.15573 (11)	0.0604 (7)	
H9	0.008157	0.161105	0.171555	0.072*	
C17	−0.3164 (2)	1.2347 (4)	0.25798 (10)	0.0554 (6)	
C10	−0.0593 (2)	0.4380 (4)	0.18025 (11)	0.0633 (7)	
H10	−0.103039	0.413615	0.212520	0.076*	
C4	0.3354 (3)	−0.3611 (4)	−0.02258 (13)	0.0730 (8)	
H4	0.368326	−0.480573	−0.034954	0.088*	
C2	0.1815 (2)	−0.1174 (4)	−0.02580 (11)	0.0591 (6)	
H2	0.108577	−0.073664	−0.040601	0.071*	
C3	0.2304 (3)	−0.2947 (4)	−0.04399 (12)	0.0685 (7)	
H3	0.190696	−0.368653	−0.071115	0.082*	
C6	0.3456 (2)	−0.0741 (4)	0.03558 (12)	0.0748 (8)	
H6	0.385856	−0.001037	0.062720	0.090*	
C21	−0.2771 (3)	1.3214 (5)	0.31308 (12)	0.0845 (9)	
H21A	−0.233080	1.442446	0.306791	0.127*	
H21B	−0.226367	1.227583	0.332118	0.127*	
H21C	−0.346982	1.349204	0.335350	0.127*	
C22	−0.5451 (3)	1.3542 (5)	0.14498 (13)	0.0852 (9)	
H22A	−0.541242	1.316139	0.106561	0.128*	
H22B	−0.535005	1.495758	0.148101	0.128*	
H22C	−0.622139	1.316783	0.159984	0.128*	
C5	0.3934 (3)	−0.2527 (5)	0.01725 (16)	0.0882 (10)	
H5	0.465538	−0.299204	0.032209	0.106*	

### 4-(Benzyloxy)phenyl 3,5-dimethylbenzoate Atomic displacement parameters (Å^2^)

**Table:**

	*U*^11^	*U*^22^	*U*^33^	*U*^12^	*U*^13^	*U*^23^
O1	0.0695 (11)	0.0396 (8)	0.0621 (10)	0.0148 (8)	0.0202 (8)	0.0071 (7)
O2	0.0578 (10)	0.0521 (10)	0.0725 (11)	0.0141 (8)	0.0035 (8)	−0.0158 (8)
C1	0.0496 (12)	0.0438 (12)	0.0505 (13)	0.0078 (10)	0.0111 (10)	0.0071 (10)
C16	0.0492 (13)	0.0510 (13)	0.0558 (14)	0.0007 (10)	0.0072 (11)	−0.0004 (11)
C15	0.0487 (12)	0.0392 (12)	0.0560 (14)	−0.0011 (10)	0.0126 (10)	−0.0035 (10)
C11	0.0495 (13)	0.0428 (12)	0.0652 (15)	0.0054 (10)	0.0071 (11)	−0.0045 (11)
C8	0.0498 (12)	0.0395 (12)	0.0558 (14)	0.0060 (10)	0.0059 (10)	0.0019 (10)
C7	0.0681 (15)	0.0461 (13)	0.0555 (14)	0.0099 (11)	0.0134 (12)	0.0070 (11)
C20	0.0562 (14)	0.0512 (13)	0.0520 (14)	0.0035 (11)	0.0090 (11)	−0.0069 (11)
C12	0.0624 (15)	0.0373 (12)	0.0685 (16)	0.0083 (11)	0.0059 (12)	0.0046 (11)
O3	0.0809 (13)	0.0679 (12)	0.0962 (15)	0.0187 (10)	−0.0142 (11)	−0.0380 (11)
C18	0.0648 (15)	0.0460 (13)	0.0600 (15)	0.0054 (11)	0.0183 (12)	−0.0091 (11)
C14	0.0584 (15)	0.0413 (12)	0.0639 (16)	0.0020 (11)	0.0066 (12)	−0.0067 (11)
C13	0.0571 (14)	0.0416 (12)	0.0626 (15)	0.0050 (11)	0.0142 (11)	0.0060 (11)
C19	0.0582 (14)	0.0504 (13)	0.0585 (15)	0.0095 (11)	0.0127 (12)	−0.0001 (11)
C9	0.0732 (17)	0.0397 (12)	0.0683 (16)	0.0075 (11)	0.0206 (13)	0.0094 (11)
C17	0.0565 (14)	0.0524 (13)	0.0573 (15)	−0.0036 (11)	0.0123 (11)	−0.0110 (11)
C10	0.0714 (17)	0.0544 (15)	0.0641 (16)	0.0019 (12)	0.0237 (13)	0.0008 (12)
C4	0.082 (2)	0.0497 (15)	0.088 (2)	0.0157 (14)	0.0367 (17)	0.0043 (15)
C2	0.0549 (14)	0.0546 (14)	0.0678 (17)	0.0105 (12)	−0.0004 (12)	0.0033 (12)
C3	0.0793 (19)	0.0535 (15)	0.0726 (18)	0.0050 (14)	0.0118 (15)	−0.0069 (13)
C6	0.0670 (17)	0.0733 (19)	0.084 (2)	0.0199 (15)	−0.0146 (15)	−0.0043 (15)
C21	0.090 (2)	0.091 (2)	0.073 (2)	0.0045 (17)	−0.0010 (16)	−0.0323 (17)
C22	0.091 (2)	0.086 (2)	0.079 (2)	0.0381 (18)	0.0006 (17)	−0.0072 (17)
C5	0.0646 (19)	0.083 (2)	0.117 (3)	0.0370 (17)	−0.0005 (18)	0.015 (2)

### 4-(Benzyloxy)phenyl 3,5-dimethylbenzoate Geometric parameters (Å, º)

**Table:**

O1—C8	1.376 (2)	C18—C19	1.385 (3)
O1—C7	1.426 (3)	C18—H18	0.9300
O2—C14	1.363 (3)	C13—H13	0.9300
O2—C11	1.415 (3)	C19—C22	1.509 (4)
C1—C6	1.373 (3)	C9—C10	1.383 (3)
C1—C2	1.376 (3)	C9—H9	0.9300
C1—C7	1.501 (3)	C17—C21	1.511 (3)
C16—C17	1.385 (3)	C10—H10	0.9300
C16—C15	1.386 (3)	C4—C3	1.348 (4)
C16—H16	0.9300	C4—C5	1.363 (4)
C15—C20	1.383 (3)	C4—H4	0.9300
C15—C14	1.485 (3)	C2—C3	1.379 (3)
C11—C12	1.363 (3)	C2—H2	0.9300
C11—C10	1.373 (3)	C3—H3	0.9300
C8—C9	1.381 (3)	C6—C5	1.383 (4)
C8—C13	1.383 (3)	C6—H6	0.9300
C7—H7A	0.9700	C21—H21A	0.9600
C7—H7B	0.9700	C21—H21B	0.9600
C20—C19	1.382 (3)	C21—H21C	0.9600
C20—H20	0.9300	C22—H22A	0.9600
C12—C13	1.385 (3)	C22—H22B	0.9600
C12—H12	0.9300	C22—H22C	0.9600
O3—C14	1.192 (3)	C5—H5	0.9300
C18—C17	1.379 (3)		
			
C8—O1—C7	117.06 (16)	C20—C19—C22	121.4 (2)
C14—O2—C11	115.93 (18)	C18—C19—C22	120.6 (2)
C6—C1—C2	118.1 (2)	C8—C9—C10	120.2 (2)
C6—C1—C7	120.5 (2)	C8—C9—H9	119.9
C2—C1—C7	121.4 (2)	C10—C9—H9	119.9
C17—C16—C15	120.5 (2)	C18—C17—C16	118.2 (2)
C17—C16—H16	119.7	C18—C17—C21	120.4 (2)
C15—C16—H16	119.7	C16—C17—C21	121.3 (2)
C20—C15—C16	119.8 (2)	C11—C10—C9	119.3 (2)
C20—C15—C14	117.3 (2)	C11—C10—H10	120.3
C16—C15—C14	122.9 (2)	C9—C10—H10	120.3
C12—C11—C10	120.9 (2)	C3—C4—C5	119.8 (3)
C12—C11—O2	120.1 (2)	C3—C4—H4	120.1
C10—C11—O2	119.0 (2)	C5—C4—H4	120.1
O1—C8—C9	115.77 (19)	C1—C2—C3	121.1 (2)
O1—C8—C13	124.3 (2)	C1—C2—H2	119.5
C9—C8—C13	119.9 (2)	C3—C2—H2	119.5
O1—C7—C1	108.45 (17)	C4—C3—C2	120.2 (3)
O1—C7—H7A	110.0	C4—C3—H3	119.9
C1—C7—H7A	110.0	C2—C3—H3	119.9
O1—C7—H7B	110.0	C1—C6—C5	120.3 (3)
C1—C7—H7B	110.0	C1—C6—H6	119.8
H7A—C7—H7B	108.4	C5—C6—H6	119.8
C19—C20—C15	120.8 (2)	C17—C21—H21A	109.5
C19—C20—H20	119.6	C17—C21—H21B	109.5
C15—C20—H20	119.6	H21A—C21—H21B	109.5
C11—C12—C13	120.2 (2)	C17—C21—H21C	109.5
C11—C12—H12	119.9	H21A—C21—H21C	109.5
C13—C12—H12	119.9	H21B—C21—H21C	109.5
C17—C18—C19	122.5 (2)	C19—C22—H22A	109.5
C17—C18—H18	118.7	C19—C22—H22B	109.5
C19—C18—H18	118.7	H22A—C22—H22B	109.5
O3—C14—O2	122.9 (2)	C19—C22—H22C	109.5
O3—C14—C15	125.2 (2)	H22A—C22—H22C	109.5
O2—C14—C15	111.8 (2)	H22B—C22—H22C	109.5
C8—C13—C12	119.4 (2)	C4—C5—C6	120.5 (3)
C8—C13—H13	120.3	C4—C5—H5	119.8
C12—C13—H13	120.3	C6—C5—H5	119.8
C20—C19—C18	118.0 (2)		
			
C17—C16—C15—C20	−0.2 (3)	C15—C20—C19—C18	−1.3 (3)
C17—C16—C15—C14	177.9 (2)	C15—C20—C19—C22	177.9 (2)
C14—O2—C11—C12	81.2 (3)	C17—C18—C19—C20	0.7 (4)
C14—O2—C11—C10	−101.5 (3)	C17—C18—C19—C22	−178.6 (2)
C7—O1—C8—C9	174.7 (2)	O1—C8—C9—C10	−176.4 (2)
C7—O1—C8—C13	−3.9 (3)	C13—C8—C9—C10	2.2 (4)
C8—O1—C7—C1	−177.1 (2)	C19—C18—C17—C16	0.2 (4)
C6—C1—C7—O1	−79.6 (3)	C19—C18—C17—C21	179.0 (2)
C2—C1—C7—O1	101.1 (3)	C15—C16—C17—C18	−0.5 (3)
C16—C15—C20—C19	1.1 (3)	C15—C16—C17—C21	−179.2 (2)
C14—C15—C20—C19	−177.1 (2)	C12—C11—C10—C9	−2.6 (4)
C10—C11—C12—C13	2.9 (4)	O2—C11—C10—C9	−179.8 (2)
O2—C11—C12—C13	−179.9 (2)	C8—C9—C10—C11	0.0 (4)
C11—O2—C14—O3	2.8 (4)	C6—C1—C2—C3	−1.0 (4)
C11—O2—C14—C15	−175.36 (19)	C7—C1—C2—C3	178.3 (2)
C20—C15—C14—O3	−14.7 (4)	C5—C4—C3—C2	0.1 (4)
C16—C15—C14—O3	167.2 (3)	C1—C2—C3—C4	0.6 (4)
C20—C15—C14—O2	163.4 (2)	C2—C1—C6—C5	0.6 (4)
C16—C15—C14—O2	−14.7 (3)	C7—C1—C6—C5	−178.7 (3)
O1—C8—C13—C12	176.7 (2)	C3—C4—C5—C6	−0.5 (5)
C9—C8—C13—C12	−1.9 (4)	C1—C6—C5—C4	0.1 (5)
C11—C12—C13—C8	−0.7 (4)		

### 4-(Benzyloxy)phenyl 3,5-dimethylbenzoate Hydrogen-bond geometry (Å, º)

*Cg*1, *Cg*2 and *Cg*3 are the centroids of the C1–C6, C8–C13 and C15–C20 rings, respectively.

**Table:**

*D*—H···*A*	*D*—H	H···*A*	*D*···*A*	*D*—H···*A*
C16—H16···O2	0.93	2.47	2.764 (2)	99
C20—H20···O3	0.93	2.54	2.832 (2)	98
C3—H3···*Cg*2^i^	0.93	2.73	3.592 (3)	154
C13—H13···*Cg*1^ii^	0.93	2.98	3.794 (3)	147
C18—H18···*Cg*3^iii^	0.93	2.95	3.821 (3)	156

Symmetry codes: (i) −*x*, −*y*, −*z*; (ii) *x*, *y*+1, *z*; (iii) −*x*−1, *y*+1/2, −*z*+1/2.
